# The Salt Tax in India

**Published:** 1904-07-02

**Authors:** 


The Hospital.

A Journal of

The Medical Sciences and Hospital Administration.

Vol. XXXVI.—No. 927. JULY 2, 1904.

### The Salt Tax in India.

The versatility of Mr. Jonathan Hutchinson's genius has seldom been more conspicuously displayed than when, on Midsummer Day, he occupied the chair at a meeting of the East India Association, called together to hear a paper by Mr. Pennington in advocacy of the abandonment of the salt tax in India. Mr. Hutchinson's appearance, *dans cette galère*, was due to the expectation that he would be able, on medical and scientific grounds, to lend powerful support to the fiscal proposals about to be submitted to the meeting; and accordingly, both in opening the proceedings and in following the author of the paper, he dwelt upon the value of salt as an article of diet, under its two-fold aspect as a preservative and as a solvent of uric acid. Mr. Hutchinson entered fully into the value of salt as a preventive of urinary calculus, a malady which is extremely prevalent in India; and he explained that its action in this respect was due not only to the solvent property mentioned above, but also to the circumstances that by producing thirst it secures to the organism a larger water-supply than it might otherwise receive, and that by keeping the colloids equally diffused throughout the urine it tends to prevent the crystalline solids from agglomerating into calculi. Evidence on these points was adduced, not only from laboratory work but also from practical experience of the comparative immunity from calculus of sailors and other large consumers of salted provisions; and hence Mr. Hutchinson felt able to throw the full weight of his authority, medically and surgically speaking, upon the side of a proposal which he admitted his inability to consider adequately from the point of view of a statesman, except in so far as it might be the duty of a ruler to employ every possible means of preserving the health of his subjects. Even so, we are disposed to think that his argument was carried a little farther than it would justly bear. We have somewhere seen a book in which it is maintained that an insufficient consumption of salt is the effective cause of all diseases, and that its adequate administration would be an universal remedy; but, with all due submission to the author of this curious production, there is something to be said upon both sides of the question. Even a moderate indulgence in salted food was held by our forefathers to be among the causes of gout; and Montaigne relates how a gouty gentleman refused to follow the advice of his physicians and to abstain from ham, salted tongues, and suchlike delicacies, merely in order that he might have definite objects upon which to invoke maledictions whenever his malady assailed him. The physicians of Montaigne's time were unacquainted with the chemistry of digestion, but they were shrewd and careful observers, perfectly capable of interpreting the teachings of experience.

There is no doubt something to be said, on public grounds, for cheapening the supply of salt for special purposes, as for fish-curing and for the establishment of salt-licks for cattle; but the salt tax as a whole is not a burdensome one, and it is the only contribution made by the Indian peasantry to the support of the Government which gives them peace and security. According to the testimony of experienced Indian officials, the tax is not a subject of discontent or complaint in the country, and it is very trivial in its incidence upon individual consumption, amounting, as far as a rough comparison of weights and coinages will allow, to about 1d. in the pound. It brings in a revenue of about six millions sterling; and Mr. Pennington had no better substitute to propose for it than a sort of graduated income-tax on the whole population, rising from 6d. a year each in the case of the poorest peasant through 1s. and 1s. 6d. to higher sums in the case of the well-to-do. It does not require much argument to show that such an impost would require an army of taxgatherers to visit every hut in the country, that its collection would be enormously expensive, that partly by reason of the direct payment and partly on account of the graduation it would lead to endless heart-burning and discontent, and that it would open the door to bribery and corruption on a prodigious scale. Within the last few years, by agreements with the Native States, the manufacture of salt has been rendered a Government monopoly over the whole of India, and the duties have been equalised with great advantage to the consumer. The result has been a commensurate increase in consumption and in revenue; and there can be no doubt that the enlightened statesmen who preside over the financial affairs of our great dependency will be fully prepared to reduce the taxation of an admitted necessary of life to the point at which, by stimulating consumption, it will furnish the largest revenue, or even below that point as soon as the revenue can safely be dispensed with. In the meanwhile the aid of medical science may properly be invoked for the purpose of showing the value of salt as a condiment, and its influence in warding off certain forms of disease, but hardly as advocating crude changes in the fiscal burdens of 300,000,000 of people.

